# Testing the neutral theory of biodiversity with the microbiome dataset from cystic fibrosis patients

**DOI:** 10.1097/MD.0000000000012248

**Published:** 2018-09-14

**Authors:** Qi Huang, Yaqiang Wang, Yao Xia, Lianwei Li, Juan Luo, Shuxian Xia, Yang Sun, Yinglei Miao, Kunhua Wang, Ye Chen

**Affiliations:** a Department of Gastroenterology, State Key Laboratory of Organ Failure Research, Guangdong Provincial Key Laboratory of Gastroenterology, Guangdong Gastrointestinal Disease Research Center, Nanfang Hospital, Southern Medical University, Guangzhou; b Department of Gastroenterology, The First Affiliated Hospital of Kunming Medical University, Yunnan Institute of Digestive Disease, Kunming; c Institute of Mathematics and Information Science, Baoji University of Arts and Sciences, Baoji, Shaanxi; d Computational Biology and Medical Ecology Lab, State Key Lab of Genetic Resources and Evolution, Kunming Institute of Zoology, Chinese Academy of Sciences; e Department of General Surgery, The First Affiliated Hospital of Kunming Medical University, Yunnan Institute of digestive disease, Kunming, China.

**Keywords:** cystic fibrosis, diversity, lung microbial community, neutral theory, niche theory

## Abstract

Cystic fibrosis (CF) is a hereditary disease that is characterized by defective mucociliary clearance, airway obstruction, chronic infection, and persistent inflammation. Cystic fibrosis pulmonary exacerbation (CFPE) majorly causes the morbidity of CF patients. Although CF has been demonstrated to change the composition of lung microbial community, previous studies have not made efforts to study the differences in the mechanism of assembly and diversity maintenance of lung microbial community in CF patients. In this study, we applied the neutral theory of biodiversity to comparatively investigate the assembly and diversity maintenance of the lung microbial community before and after the antibiotic treatment by reanalyzing the dataset from Fodor et al's study. We found that no one sample in the lung microbial communities of the sputum samples of Exacerbation group, nor those of End-of-treatment group satisfied the predictions of neutral model, suggesting that the neutral-process does not dominate in CF patients before and after antibiotic treatments. By comparing the biodiversity parameter between Exacerbation and End-of-treatment group, we found that the former had the significantly higher biodiversity, but the change in diversity parameter is slight and the *P* value is close to.05 (*P* value = .41). Therefore, our second finding is that although CFPE may increase the biodiversity of lung microbial community, the change is not essential.

## 1 Introduction

Cystic fibrosis (CF) arises from the mutation of a gene encoding the functional protein, cystic fibrosis transmembrane conductance regulator (*CFTR*) which is involved in the production of sweat, digestive fluids, and mucus.^[1]^ The *CFTR* protein plays important role in mucosal surfaces, and its dysfunction usually gives rise to thicker secretion of glands, therefore results in obstruction of exocrine glands all over the body. CF is a multisystem disease and influences many body parts, such as lung, pancrea, liver, kidney, and intestine, among which lung is most heavily affected. It is considered one of the most widespread genetic disorders shortening human life expectancy. CF is the most common lethal genetic disease in the people of Northern European and it is the least common in African and Asian population.^[2,3]^ The most typical symptoms of CF are chronic pulmonary obstruction, pancreatic dysfunction, and abnormal increased level of electrolytes (i.e., sodium and fluorine) in sweat. The long-term results of CF include recurrent pulmonary infection, dyspnea, and mucous sputum. The other CF complications are sinus infection, poor growth, fatty stool, clubbed-fingers, and male infertility. Although CF patients are living longer thanks to the improvements in management of airway infections, it is still incurable, and the extra-pulmonary complications from CF, such as CF-related diabetes, kidney disease, osteoporosis, depression, and arthropathy, are becoming increasing threats.

Several studies have shown that both long- and short-term fluctuations in lung function can be related to disease severity due to *CFTR* gene mutations, bacterial infection, and periodic pulmonary exacerbation.^[1]^ The main colonized bacterial species include *Pseudomonas aeruginosa, Staphylococcus aureus, Prevotella, Achromobacter xylosoxidans, Veillonella, Ralstonia, Rothia,* and *Haemophilus influenzae*.^[4–6]^ In addition to bacteria, fungi are detected in a large number of CF patients, including *Aspergillus fumigatus, Aspergillus terreus,* and *Scedosporium species for filamentous fungi*.^[7]^ The candidaemia (e.g., *Candida parapsilosis* and *Candida albicans*) are also found more commonly in frequent CFPE).^[8]^ Meanwhile, respiratory viral infection may be an aggravated event and some studies show that about a third of CF exacerbations are associated with viruses infection.^[9]^ RNA viruses (e.g., coronavirus, rhinovirus, parainfluenzae, etc.) infections can significantly trigger the period of CFPE.^[10–13]^

In previous studies, it has been found that the microbial community of lung could be changed due to alerted microenvironment of lung, which may result from diseases or external environmental disturbance.^[4,14–23]^ In the case of CF, mutations in *CFTR* can change the ion flux, impact the normal airway function, and compromise the normal innate immune defense finally, making the lower airways easier to be infected. Although typically only a few species are associated with chronic airway infections in CF, along with the improved treatments of infections, new species associated with CF has emerged, and the total microbes should be took into account as a whole. After infections, numerous species in lung form a microbial community, and the common-used antibiotic treatment bring a selective process, leading to significant changes of the composition of community. From an ecologist's view, the lung infected by microbes could be seen an ecosystem colonized by microbes, where the interactions between individuals or species and between microbes and environment play an important role. Therefore, variant ecological viewpoints and methods could be used to study the lung microbial community and its changes.

One core topic of community ecology is the mechanism of community assembly and diversity maintenance, which is our choice to revealing the influence of CF on lung microbial community in this study. Traditionally, there are 2 major theories for explaining the mechanism, the niche theory emphasizing the deterministic factors and the neutral theory emphasizing the stochastic factors. The Niche theory has dominated in this field for a long time till Hubbell et al^[24]^ proposed the neutral theory, which is characterized by the assumption that individuals from 1 community composed of different species at the same trophic level are completely equal, species migrate randomly, and the number of species depends on the balance between extinction and immigration or new species formation.^[25–27]^ The neutral theory combines neutrality, stochasticity, sampling and dispersal, and provides a null model to test the neutrality of a community and a simple mechanistic explanation of the species abundance distributions (SAD), based on which it is possible to decide, accept, or reject specific hypothesis regarding the mechanism underlying community assembly and diversity maintenance. For these merits, neutral theory has been widely adopted in macroecology area for a long time,^[28,29]^ and been an effective tool in microecology area recently thanks to the development of the next generation DNA sequencing (NGS) technology.^[30–40]^ A previous study has revealed that in the lungs of healthy individuals, neutral processes override selective processes, indicating the species in lungs are neutrally distributed.^[36]^ However, in CF patients, especially those who have pulmonary exacerbation, the lung microbial community could be totally different due to altered microenvironment. After antibiotic treatments, the lung microbial communities of CF patients are expected to restore back to the original healthy state as far as possible. Therefore, in this study, our aim is to understand whether or not the neutral process would dominate in the lung microbial community of CF patient, and whether antibiotic treatments could revert it to another state. To answer these questions, we apply 2 widely used sampling models proposed by Ewens ^[41]^ and Etienne ^[42]^ for testing Hubbell neutral theory to reanalyze the dataset from Fodor et al's^[43]^ study.

## 2 Methods and material

### 2.1 Ethics statement

The dataset we used to test the neutral theory was from a study performed by Fodor et al.^[43]^ This study was conducted with the approval of the Office for Research Ethics Northern Ireland and all study participants provided informed written consent.

### 2.2 Dataset description

Fodor et al collected spontaneous expectorated sputum samples and mouthwash samples from 23 adult CF patients at 2 time points, one of which is the beginning of an acute exacerbation, and another is after antibiotic treatment for the exacerbation. Total DNA was extracted, and V1-V2 region of 16S rRNA gene was amplified and sequenced using 454 pyrosequencing to identify the species of microbial communities in these samples. The average number of sequences per sample was approximately 4300. A total of 169 operational taxonomic units (OTUs) at a 97% similarity level were identified. In our study, we picked 25 sputum samples at the onset of a clinically diagnosed exacerbation (defined as Exacerbation Group) and 25 sputum samples at the End-of-treatment (defined as End-of-treatment Group) to test the neutral theory regarding microbial communities.

### 2.3 Calculation and analysis method

Ewens^[41]^ and Etienne^[42]^ sampling formula were used as the sampling models in this paper. Several studies have adopted these models to test the assembly and diversity maintenance mechanism of human microbial community.^[38–40]^ Ewens sampling formula was put forward by Warren Ewens in 1972 for describing the differences in alleles.^[41]^ In 2001, it was introduced into ecology area by Stephen Hubbell for calculating the likelihood of the presence of an ecological community consisting of *S* species with abundance of *n*_*1*_*, n*_*2*_*, n*_*3*_*... n*_*s*_ and measuring its consistency with the prediction of neutral theory. The equation is given as follows: 
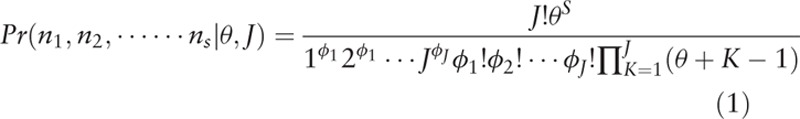


where, *J* represents the number of individuals in the community, *ϕ*_*a*_ expresses the number of species with abundance *a*, and *n*_*i*_ is the abundance of the species *I*, and *θ* denotes the biodiversity parameter with the following definition: 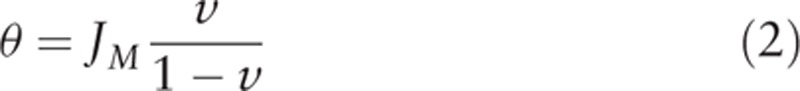


where *J*_*M*_ represents the number of individuals in the metacommunity and *v* is the per capita speciation rate. *θ* could be estimated using maximum likelihood estimation.

If a community satisfies the neutral prediction, species in this community must have dispersal limitation. However, the effect of dispersal limitation is not taken into consideration in the Ewens sampling formula. Etienne introduced a new sampling model that considers the limited dispersal factor, which is defined as: 
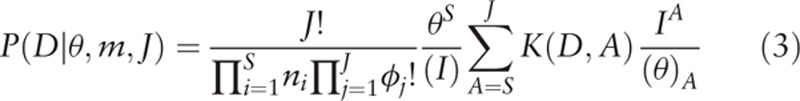


where *m* is the immigration rate and defined as: 
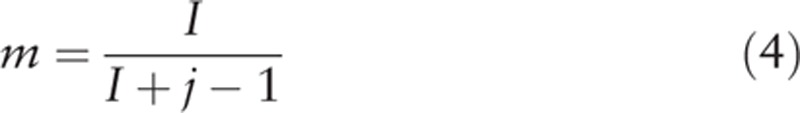


and *K*(*D*, *A*) is defined as: 
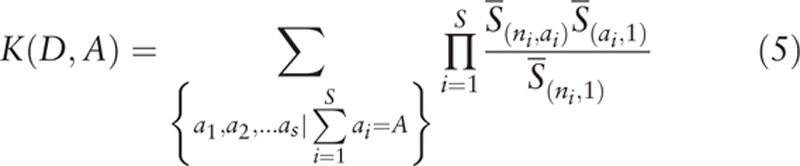


and other parameters have the same definitions in the Ewens sampling formula. In fact, Ewens sampling formula can be viewed as a specific state of Etienne model with unlimited dispersal of individuals (*m* *=* *1*).

We adopted an exact neutrality test method^[44]^ to test the statistical fitness of the neutral theory model to the dataset (for short, neutrality) in both Ewens and Etienne formula. In brief, we used the maximum likelihood estimation (MLE) for estimating the parameters based on the observed samples firstly with the R package *untb* (available at: https://cran.rproject.org/web/packages/untb/index.html). Secondly, for each sample, 100 artificial datasets were simulated with the parameters (*θ, I, J*) which are estimated via observed samples. Then we used Etienne formula to calculate the likelihood for each artificial dataset (*L*_*0*_) and corresponding observed sample (*L*_*1*_). Finally, we compared *L*_*0*_ and *L*_*1*_ using a *χ*^2^test in the following equation: 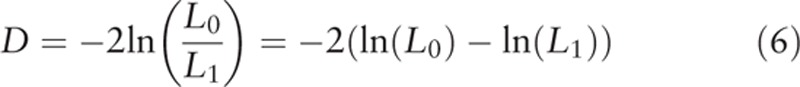


The null hypothesis is that there is no significant difference between the likelihood of the artificial dataset (*L*_*0*_) and corresponding observed sample (*L*_*1*_). If no significant difference was found (*P* value > .5), the microbial community is considered to satisfy the prediction of neutral theory.

## 3 Results and discussion

### 3.1 Diversity and neutrality exact test in CF patients

Ewens and Etienne sampling formulae were respectively used to calculate whether the microbial communities of individuals in Exacerbation Group satisfied the neutral theory prediction. The detailed results were displayed in Tables 1 and 2, with Table 1 displaying the results produced by Ewens sampling formula and Table 2 presenting the results produced by Etienne sampling formula. In the tables, ID is the sample code, *J* represents the number of microbial individuals in the sample, *S* is the number of microbial species, *θ* denotes the biodiversity parameter, *m* is the immigration rate of species, Log(L_*0*_) expresses the likelihood of a microbial community, and Log(L_1_) stands for the simulated community likelihood. The *q*-value represents the difference of likelihoods in the observed and simulated microbial communities, which is defined as the *P* value. The mean values of diversity parameters *θ* for Exacerbation Group calculated using Ewens and Etienne formulae are 6.2495 and 6.314 respectively. In most cases in the Etienne result, the immigrate rate *m* is very close to 1, indicating that the dispersal limitation is not in effect for most samples. As for the neutrality test, none of samples satisfied the prediction of neutral theory with both sampling formulae.

**Table 1:**
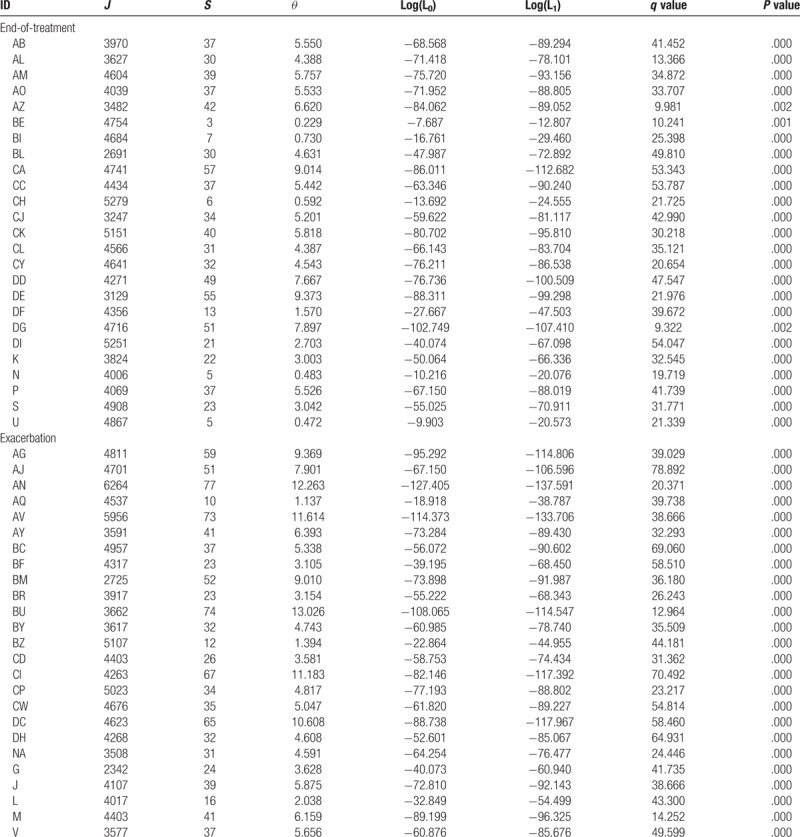
The full results of neutral theory testing with sputum microbiome using Ewens sampling formula.

**Table 2:**
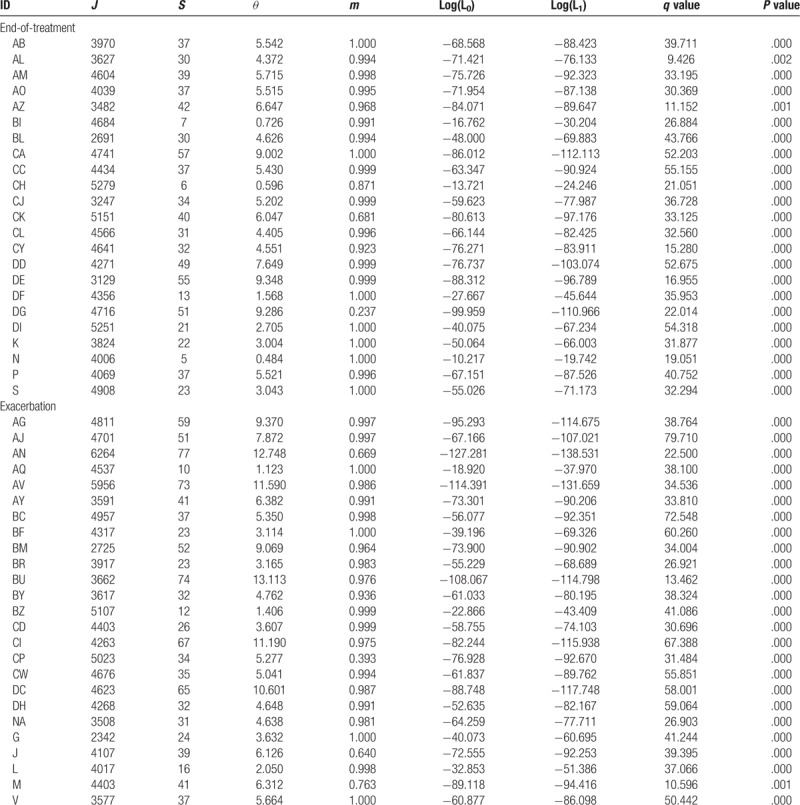
The results of neutral theory testing with sputum microbiome using Etienne sampling formula.

### 3.2 Change of diversity and neutrality after antibiotic treatment

**Figure 1:**
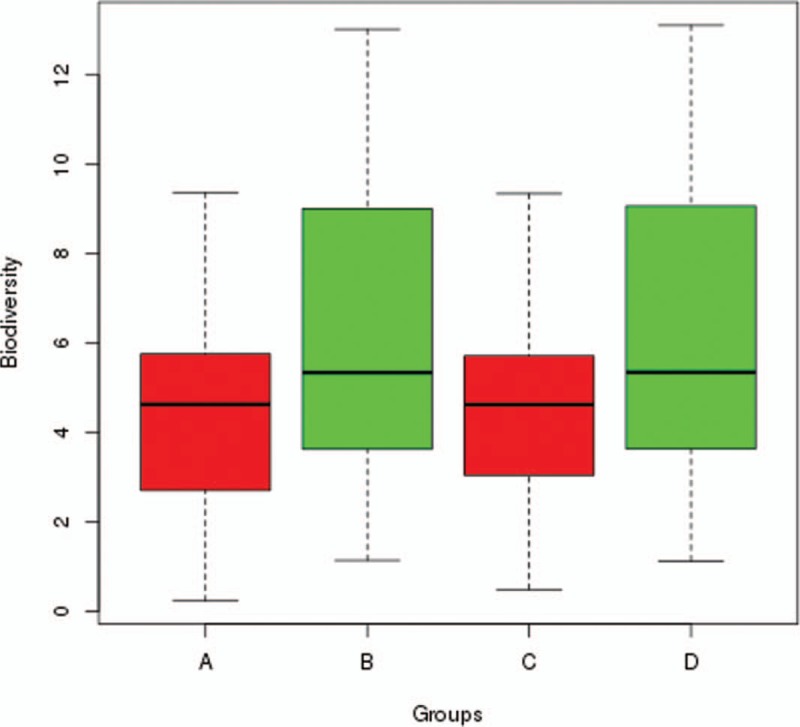
Differences in fundamental diversity parameters *θ*. A and B are the fundamental diversity parameters *θ* of End-of-treatment group and Exacerbation group respectively calculated according to Ewens sampling formula, while C and D are the ones respectively calculated according to Etienne sampling formula.

Ewens and Etienne sampling formulae were used for calculating diversity and testing neutrality for End-of-treatment Group as well. The mean values of diversity parameters *θ* for End-of-treatment Group calculated using Ewens and Etienne formulae are 4.407 and 4.825 respectively. Using the Etienne formulae, 2 samples in End-of-treatment Group cannot be calculated because there are too few individuals and species. In addition, the immigrate rate *m* of most samples in the results of Etienne formula is very close to 1, implying that Ewens and Etienne sampling formulae perform similarly for this dataset. Therefore, we chose the results of Ewens formula for the statistical analysis. We compare the diversity parameters of End-of-treatment Group and Exacerbation Group (Fig. 1) using a T test, and found there is significant difference between 2 groups (*P* value = .041), suggesting that antibiotic treatment could significantly decrease the biodiversity of lung microbial community. However, no one sample was found to satisfy the prediction of neutral theory, which meant that after antibiotic treatment, the mechanisms of community assembly and diversity maintenance of lung microbial communities are dominated by Niche process.

## 4 Discussion

The lung is considered effectively sterile via innate immune mechanisms in healthy state, hence less microbes could colonize in this body part comparing with other parts such as gut, oral, etc. Although the lung is connected to the oral and nasal cavity directly, the amount of microbes in healthy lung is much lower (the quality of DNA of total microbes in healthy lung is 3 orders of magnitude less than that from the oral).^[36]^ Given these factors, the microbial community of lung should be quite different in many aspects comparing with those of other body parts. Immigration from upper airway is an important driver shaping the community, and even slight change in lung caused by lungs diseases or external environmental factors may bring significant change in lung microbial community. While it is hard to predict precisely how the changes in lungs influence the lung microbial community, plenty of studies have shown such changes. For instance, Yu et al^[22]^ reported that the lung tissue microbiota showed increased alpha diversity with environmental exposures such as air particulates, residence in high population density areas, and pack-years of tobacco smoking. Krishna et al^[23]^ showed the presence of diverse opportunistic pathogenic microbiota in TB patients increased the complexity and diversity of sputum microbiota. Paganin et al^[20]^ detected that changes of microbial community in CF airway were relevant to a severe decline of lung function. Price et al^[4]^ found that the community diversity cannot distinguish the clinical stages in CF. Cuthbertson et al^[21]^ suggested that core microbiota members did not have significant negative impact on the CFPE. Though the changes in composition of microbial community have been demonstrated widely, few studies have shed light on the key question: how is the force shaping lung microbial community affected by the alterations in lung?

The neutral theory underlying the stochastic factors such as immigration, birth/death, and speciation is an alternative for the classical niche theory underlining the deterministic interspecies and species-environment interactions. To test if the SAD curves of a certain community fit the prediction of neutral theory is an effective and simple way to investigate the mechanisms of community assembly preliminarily. Not only in macroecology area, this method has been used in microecology area. Arvind et al^[21,36]^ demonstrated that, in healthy lungs, the composition of microbial communities satisfied the predictions of neutral model, and no one specie deviated from the model expectations. Their results suggest that the neutral process is the main force shaping the healthy lung microbial community. In the microbial community of healthy lung, most invading microbes are eliminated via innate immune mechanisms, and the remaining ones may have strong enough resistance against the environmental factors that can remove them. Therefore all the remaining species in the community may be ecologically equal (i.e., they have equal chance to immigrate, growth, and death). In the microbial community of CF patient, however, things have changed. Most importantly, the normal innate immune mechanism of lung has changed due to the mutations in *CFTR*, resulting in significant alterations in the microenvironment of lung. The change of composition of lung community is not evitable, and such change may be driven by the environmental factors or certain species.^[45]^ This raises a question: in the CF patient, whether or not the neutral process is still the dominant force that shapes the lung microbial community? In our study, we used Ewens and Etienne sampling formulae to test if the lung microbial communities in CF patients (Exacerbation Group) satisfied the predictions of neutral theory, and found no one sample satisfied the predictions with both methods, suggesting that, unlike the healthy lung, the assembly of lung microbial communities in CF patients may not be dominated by neutral process any more. This result suggests that CF would change not only the composition, but also the assembly mechanism of lung microbial community.

In CF patients, the stable disease symptoms are disrupted by the episodes of acute pulmonary exacerbation, and can change the composition of lung community significantly. Treatments for CF mainly include antibiotics along with pancreatic enzyme replacement, fat-soluble vitamin supplement, or lung transplantation. Usually, patients would be subjected to repeat rounds of antibiotics for controlling the infections. The lung microbial communities may have likely evolved and adapted in response to antibiotic treatments, which may lead very different compositions of microbial communities. In Fodor et al's study, they surprisingly found that most patients were treated for 2 weeks with a combination of broad-spectrum antibiotics, but the lung microbial community composition did not have substantively change. Because although small decrease was detected in richness after antibiotic treatments, the changes occur in only taxa with low relative abundance and the most abundant taxa in this cohort showed little change in response to antibiotics. The observation may result from the fact that antibiotic resistance is widespread throughout plenty of abundant species. In our study, similar result was detected by comparing the diversity parameter *θ* between End-of-treatment Group and Exacerbation Group. We found that while the significant was detected between End-of-treatment and Exacerbation Group, the mean value of *θ* in End-of-treatment is slightly lower than Exacerbation Group, and the *P* value is very close to.05, which indicates that the significance is not so strong. This result raises another key question: after antibiotic treatment, whether or not the mechanism of assembly of lung microbial community would change back to the state in healthy lung, which is dominated by neutral process. We tested the neutrality in the samples from CF patients who have received 2 weeks treatments of antibiotic (End-of-treatment Group) with Ewens and Etienne sampling formulae, and found that no one satisfied the predictions of neutral theory, indicating that the antibiotic treatment may not change the mechanism of assembly of lung microbial community back to the neutral process dominating state.

There are 2 main limitations in our study. First, the chosen models treat the neutral- and niche-driven community as a dichotomy, which may not be rigorous enough as there may be a mediacy between them. Hence, more sophisticated hybrid models that consider neutral- and niche-process as a continuum could be used in further studies to improve our results.^[46–49]^ Nevertheless, given that traditional neutral and niche process are the 2 ends of the neutral-niche continuum and the aim of our study is trying to investigate the lung microbial community assembly linking to CF preliminarily, our results do not contradict the further studies we proposed, and provide the first piece of evidence to demonstrate the CF-related change in the assembly mechanisms of lung microbial community instead. Second, although SAD-fitting models have been the most widely used methods for investigating the community assembly, which is used in our study as well, they have limitations (e.g., this method can hardly distinguish contributions of sampling effects and other more ecologically based effects).^[50,51]^ Further studies could try to use models that considering SAD with other information simultaneously (e.g., identity of species and phylogenetic information) to understand the assembly mechanism of the lung microbial community related to CF better.

## Author contributions

**Conceptualization:** Ye Chen.

**Data curation:** Yaqiang Wang.

**Formal analysis:** Qi Huang, Yao Xia, Lianwei Li.

**Investigation:** Qi Huang.

**Methodology:** Yao Xia, Ye Chen.

**Supervision:** Shuxian Xia, Kunhua Wang, Ye Chen.

**Validation:** Yao Xia, Lianwei Li.

**Visualization:** Yaqiang Wang, Yao Xia, Lianwei Li.

**Writing – original draft:** Qi Huang, Yao Xia.

**Writing – review & editing:** Juan Luo, Shuxian Xia, Yang Sun, Yinglei Miao, Kunhua Wang, Ye Chen.
